# Development of ultra-short PCR assay to reveal *BRAF* V600 mutation status in Thai colorectal cancer tissues

**DOI:** 10.1371/journal.pone.0198795

**Published:** 2018-06-07

**Authors:** Nunthawut Chat-Uthai, Pichpisith Vejvisithsakul, Sutthirat Udommethaporn, Puttarakun Meesiri, Chetiya Danthanawanit, Yannawan Wongchai, Chinachote Teerapakpinyo, Shanop Shuangshoti, Naravat Poungvarin

**Affiliations:** 1 Research Division, Faculty of Medicine Siriraj Hospital, Mahidol University, Bangkok, Thailand; 2 Clinical Molecular Pathology Laboratory, Department of Clinical Pathology, Faculty of Medicine Siriraj Hospital, Mahidol University, Bangkok, Thailand; 3 Chulalongkorn GenePRO Center, Research Affairs, Faculty of Medicine, Chulalongkorn University, Bangkok, Thailand; 4 Department of Pathology, Faculty of Medicine, Chulalongkorn University, Bangkok, Thailand; University of Crete, GREECE

## Abstract

The protein kinase BRAF is one of the key players in regulating cellular responses to extracellular signals. Somatic mutations of the *BRAF* gene, causing constitutive activation of BRAF, have been found in various types of human cancers such as malignant melanoma, and colorectal cancer. BRAF V600E and V600K, most commonly observed mutations in these cancers, may predict response to targeted therapies. Many techniques suffer from a lack of diagnostic sensitivity in mutation analysis in clinical samples with a low cancer cell percentage or poor-quality fragmented DNA. Here we present allele-specific real-time PCR assay for amplifying 35- to 45-base target sequences in *BRAF* gene. Forward primer designed for BRAF V600E detection is capable of recognizing both types of BRAF V600E mutation, i.e. V600E1 (c.1799T>A) and V600E2 (c.1799_1800delTGinsAA), as well as complex tandem mutation caused by nucleotide changes in codons 600 and 601. We utilized this assay to analyze Thai formalin-fixed paraffin-embedded tissues. Forty-eight percent of 178 Thai colorectal cancer tissues has *KRAS* mutation detected by highly sensitive commercial assays. Although these DNA samples contain low overall yield of amplifiable DNA, our newly-developed assay successfully revealed BRAF V600 mutations in 6 of 93 formalin-fixed paraffin-embedded colorectal cancer tissues which *KRAS* mutation was not detected. Ultra-short PCR assay with forward mutation-specific primers is potentially useful to detect BRAF V600 mutations in highly fragmented DNA specimens from cancer patients.

## Introduction

Human v-raf murine sarcoma viral oncogene homologue B1 gene (*BRAF*), is located on chromosome 7q34. The cytoplasmic serine-threonine kinase encoded by this gene mediates the activation of the mitogen-activated protein kinase (MAPK) signaling pathway involved in cell growth, survival and differentiation. About half of nonacral cutaneous melanomas harbor gain-of-function *BRAF* mutations, rendering the MAPK pathway constitutively active [1]. The most common mutations in *BRAF* occur in codon 600. The majority of the *BRAF* V600 mutations are V600E [2], results in an amino acid substitution at position 600 in BRAF, from a valine (V) to a glutamic acid (E). The next most commonly observed *BRAF* mutations are V600K, which arises from a double nucleotide change and results in an amino acid substitution of the valine (V) at position 600 by a lysine (K). These mutations account for approximately 95% of *BRAF* mutations found in melanoma [3]. Other mutations, including V600M, V600R, V600D and V600G, are less common. Mutations of *BRAF* gene can also be found in colorectal cancer (CRC), papillary thyroid cancer, lung cancer and hairy cell leukemia [4]. Patients with *BRAF*-mutated CRC have a poor prognosis [5]. Frequency of *BRAF* mutations in CRC varies widely among different populations around the world [6–11]. The overall frequency of the CRC with BRAF V600 mutations in Asian populations is relatively low in comparison to other ethnic populations [12].

Molecular identification of mutation status has become important part of precision medicine. BRAF inhibitors have demonstrated impressive clinical activity in patients with advanced melanoma that contains the activating *BRAF* V600 mutations [13–16]. Vemurafenib, Dabrafenib, Trametinib and Cobimetinib were approved by the US Food and Drug Administration (FDA) for the treatment of these patients [9, 17–19]. Treatment responses induced by BRAF inhibitors in *BRAF* V600E-mutated lung adenocarcinoma have also been reported [20, 21]. In CRC, most patients without mutated Kirsten rat sarcoma viral oncogene homolog gene (*KRAS)* in their tumors respond to treatment by anti-EGFR monoclonal antibodies panitumumab and cetuximab. Mutations in *BRAF* gene have been implicated for unresponsiveness to EGFR inhibitor therapy in a small but significant proportion of CRC patients without *KRAS* mutations [22].

Many polymerase chain reaction (PCR)-based techniques, including traditional bidirectional direct (Sanger) sequencing, pyrosequencing, high-resolution melting analysis [23, 24], PCR clamping [25] and allele-specific PCR (AS-PCR) [26], have been utilized to detect these *BRAF* mutations in human cancer specimens. Level of validation of these tests is highly variable. Most of these PCR-based assays have been designed to work using good quality DNA. In real-life, poor-quality DNA obtained from formalin-fixed paraffin embedded (FFPE) tissues can be problematic. FFPE DNA is often highly cross-linked, and fragmented. The DNA of these samples can be fragmented down to less than 100 bp fragment lengths [27–29]. A number of techniques suffer from extensively fragmented DNA extracted from some of these specimens which limits the amplifiability of the DNA. Moreover, endogenous and exogenous inhibitory substances, such as formalin [30] and melanin [31], may be present in DNA samples. Intra- and inter-tumor heterogeneity is another challenge in determination of molecular mutation status in cancers. For all these reasons, highly sensitive assay with efficient PCR amplification and ability to target small pieces of DNA is particularly valuable for revealing minor mutant alleles hidden in wild-type background in clinical specimens.

We have previously established AS-PCR-based detection of clinically important *EGFR* mutations in exons 19, 20 and 21 [32]. In our experience working with SYBR Green I-based AS-PCR, this technique is simple, cost-effective and highly sensitive. We therefore extended the use of allele-specific quantitative PCR technique, with ability to detect minor *BRAF* mutations in extremely short DNA fragments, to uncover *BRAF* mutation status in DNA samples obtained from Thai *KRAS* mutation-negative CRC tissues in this study. This method will help us precisely identify patients who would benefit from treatment with targeted cancer therapies.

## Materials and methods

### Cell lines and genomic DNA isolation

Human embryonic kidney cell line HEK-293, containing wild-type alleles at position 1799, was cultured in DMEM culture medium (HyClone, USA) with 10% fetal bovine serum (HyClone, USA). Cells were incubated at 37°C in a 5% CO_2_ in humidified air atmosphere. We used QIAamp DNA Micro kit (Qiagen, Germany) to extract DNA from HEK-293 cells according to manufacturer's recommendation.

### Templates for assay evaluation

We designed short single-stranded oligonucleotides of 25, 35 or 45 bases, each containing various types of *BRAF* V600 mutations or wild-type *BRAF* sequences as shown in S1 Table. These oligonucleotide templates were synthesized by Macrogen (South Korea). We resuspended them in TE buffer. We diluted these stock solutions in water to prepare working solutions and used them immediately.

To determine the limit of detection (LOD) of mutation detection assays, we diluted mutation containing DNA reference standard (Horizon Diagnostics, UK) in wild-type DNA (from Horizon Diagnostics or HEK-293 cell line DNA) to 20%, 15%, 10%, 5%, 1% or 0% mutant alleles.

### Analysis of FFPE specimens

The FFPE cell block sections were provided to test the integrity of somatic mutation analysis workflow for external quality assessment (EQA) schemes in the years 2014–2016. Information on FFPE block allelic frequencies analyzed by droplet digital PCR were given by EQA provider, the European Molecular Genetics Quality Network (EMQN). The FFPE tissue blocks were sectioned using a microtome in the Department of Pathology. No microdissection was performed.

We transferred paraffin ribbons into tubes and dewaxed samples using xylene (VWR Chemicals, USA) or Deparaffinization Solution (Qiagen, Germany) and isolated DNA from these samples by cobas Sample Preparation Kit (Roche Diagnostics, Switzerland) or QIAamp DNA FFPE Tissue Kit (Qiagen, Germany). The DNA samples were then used for assessment of total amplifiable DNA by amplification of *RAG1* [33] and *RPP30* genes (S2 Table) and mutation detection.

This study was conducted under the protocols approved by Siriraj Institutional Review Board (SIRB), Faculty of Medicine Siriraj Hospital, Mahidol University (approval number Si 011/2016 and Si 496/2014). One hundred and seventy eight FFPE colorectal cancer specimens that were sent to the Clinical Molecular Pathology Laboratory, Department of Clinical Pathology, Faculty of Medicine Siriraj Hospital, Mahidol University between 2013 to 2017 for *KRAS* mutation analysis were include in this study. The data were analyzed anonymously. We excluded samples from an analysis based on the presence in duplicates, specimen type and technique used to identify mutation.

### Mutation detection

We adopted allele-specific forward primer sequence for *BRAF* mutation detection [26] and designed new tailed reverse primer. This reverse primer consists of the *BRAF* gene sequences in combination with previously published sequences [34, 35]. Oligonucleotide sequences are shown in S2 Table. These primers target 47-bp region of the *BRAF* gene. The final PCR product size is 115 nucleotides. SYBR Green-based allele-specific PCR assays were performed in previously described conditions [32] with minor modifications. Briefly, we used reaction mixture comprising 7.5μl of QuantiFAST SYBR Green PCR Kit including ROX dye (Qiagen, Germany), the total of 400 nM of *BRAF* mutant-specific primers, and 12–48 ng of genomic DNA for a 15-μl reaction. We performed touchdown PCR with SYBR Green I fluorescence monitoring on a Rotor-Gene Q (Qiagen, Germany). Post-PCR melting curve was analyzed.

We performed cobas® 4800 BRAF V600 Mutation Test (Roche Diagnostics, Switzerland) and next generation sequencing using GeneRead QIAact Actionable Insights Tumor Panel (Qiagen, Germany) according to manufacturers' recommendation. Mutations of *KRAS* gene were detected by the cobas® KRAS Mutation Test (Roche Diagnostics, Switzerland) or AmoyDx® *KRAS* Mutation Detection Kit (Amoy Diagnostics, China). The results of mutation tests were interpreted according to manufacturers' instructions.

### Statistical analysis

We performed a t-test for the comparison of the means of cycle threshold differences obtained from real-time PCR results. We used Chi-squared test or Fisher’s exact test to identify significant associations between mutation status and sex, age, and tumor location. Significance threshold was set at 0.05.

## Results

### Allele-specific PCR identifies the existence of single nucleotide substitution and complex tandem *BRAF* mutants

**Fig 1:**
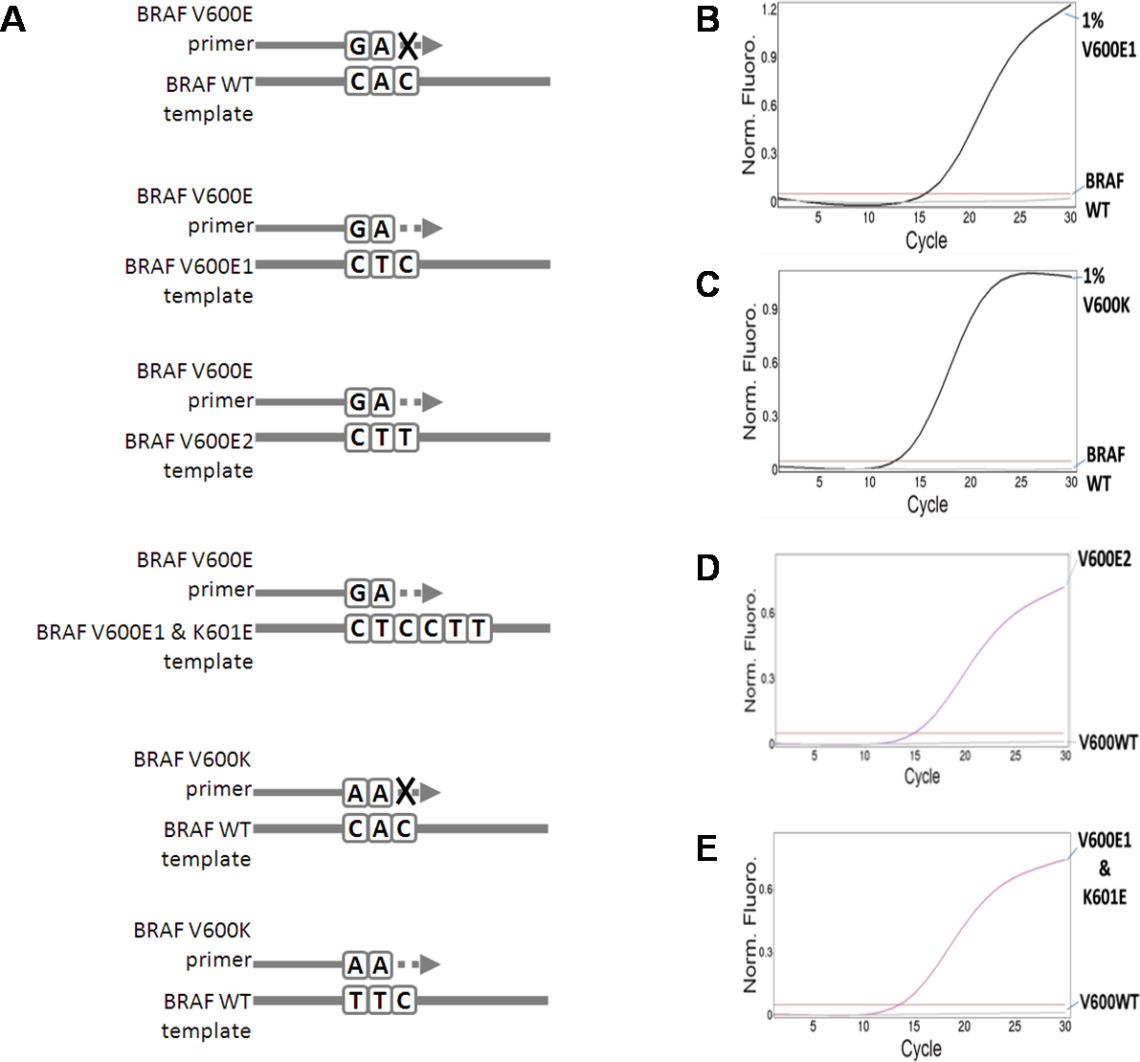
Allele-specific forward primers allow detection of single and double *BRAF* mutations. A. Schematic diagrams of primer design. Forward primers were chosen to specifically amplify BRAF V600E1 (c.1799T>A), V600E2 (c.1799_1800delTGinsAA) and V600K (c.1798_1799delGTinsAA) mutations. Detection of BRAF V600K mutation can be performed separately or together with BRAF V600E mutation detection. WT = wild-type. B and C. Detection of low abundant BRAF V600E1 (B) and V600K mutations (C) in diluted DNA reference standards, which allelic frequencies were measured by droplet digital PCR. DNA amplification is monitored at each cycle of PCR using SYBR Green reagent included in PCR premix. D and E. Detection of BRAF V600E2 mutation (D) and complex tandem mutation caused by nucleotide changes in codons 600 and 601 (BRAF V600E1 and K601E mutations) (E). Synthesized oligonucleotides (S1 Table) were diluted and used as PCR templates.

We first developed assay to detect common BRAF V600 mutations, i.e. V600E (c.1799T>A or c.1799_1800delTGinsAA) and V600K (c.1798_1799delGTinsAA), which indicate a response to cancer treatment by BRAF inhibitors [13–16]. This assay must be easy to perform and sensitive enough to accurately detect very low abundant somatic mutations. We chose allele-specific quantitative PCR method for our assay development. The design of allele-specific forward primer sequences was adopted from previous publication [26]. This design (Fig 1A) will allow us to identify BRAF V600E1, V600E2 mutations and either BRAF V600E1 or V600E2 mutation that co-exists with mutations in codon 601.

Our assays performed well and detected 1% alleles of *BRAF* c.1799T>A mutation (BRAF V600E1) (Fig 1B and Table 1) or *BRAF* c.1798_1799delGTinsAA (BRAF V600K) (Fig 1C) in DNA reference standards. Due to unavailability of DNA reference standard, we used synthesized oligonucleotides containing *BRAF* c.1799_1800delTGinsAA mutation (BRAF V600E2) or *BRAF* c.[1799T>A(;)1801A>G], complex tandem *BRAF* mutations, as PCR template. We demonstrate that our assay can detect these mutations presented in the oligonucleotide templates (Fig 1D and 1E). This design of *BRAF* mutation assay with mutation-specific forward primers is therefore useful to identify clinically significant single mutations as well as complex tandem mutations.

**Table 1:** Detection of BRAF V600E mutations in reference standards by AS-PCR assays.

Sample number	Specimen type	*BRAF* genotype (% allelic frequency) by droplet digital PCR assay	*BRAF* genotype by AS-PCR assay	*BRAF* genotype by cobas® 4800 BRAF V600 Mutation Test
**1**	DNA	No mutation present	Mutation not detected	Mutation not detected
**2**	DNA	c.1799T>A p.(V600E) (1%)	V600E mutation detected	Mutation not detected
**3**	DNA	c.1799T>A p.(V600E) (5%)	V600E mutation detected	Mutation not detected
**4**	DNA	c.1799T>A p.(V600E) (10%)	V600E mutation detected	Mutation not detected
**5**	DNA	c.1799T>A p.(V600E) (15%)	V600E mutation detected	V600 mutation detected
**6**	DNA	c.1799T>A p.(V600E) (20%)	V600E mutation detected	V600 mutation detected
**7**	FFPE Section	No mutation present	Mutation not detected	Mutation not detected
**8**	FFPE Section	c.1799T>A p.(V600E) (66%)	V600E mutation detected	V600 mutation detected
**9**	FFPE Section	No mutation present	Mutation not detected	Mutation not detected
**10**	FFPE Section	No mutation present	Mutation not detected	Mutation not detected
**11**	FFPE Section	No mutation present	Mutation not detected	Mutation not detected
**12**	FFPE Section	No mutation present	Mutation not detected	Mutation not detected
**13**	FFPE Section	c.1799T>A p.(V600E) (66%)	V600E mutation detected	V600 mutation detected
**14**	FFPE Section	c.1799T>A p.(V600E) (66%)	V600E mutation detected	V600 mutation detected
**15**	FFPE Section	No mutation present	Mutation not detected	Mutation not detected
**16**	FFPE Section	No mutation present	Mutation not detected	Mutation not detected
**17**	FFPE Section	No mutation present	Mutation not detected	NA
**18**	FFPE Section	c.1799T>A p.(V600E) (66%)	V600E mutation detected	NA
**19**	FFPE Section	c.1799T>A p.(V600E) (66%)	V600E mutation detected	NA
**20**	FFPE Section	No mutation present	Mutation not detected	NA
**21**	FFPE Section	c.1799T>A p.(V600E) (34%)	V600E mutation detected	NA
**22**	FFPE Section	No mutation present	Mutation not detected	NA
**23**	FFPE Section	No mutation present	Mutation not detected	NA
**24**	FFPE Section	No mutation present	Mutation not detected	NA
**25**	FFPE Section	No mutation present	Mutation not detected	NA
**26**	FFPE Section	No mutation present	Mutation not detected	NA
**27**	FFPE Section	No mutation present	Mutation not detected	NA
**28**	FFPE Section	No mutation present	Mutation not detected	NA
**29**	FFPE Section	c.1799T>A p.(V600E) (65%)	V600E mutation detected	NA
**30**	FFPE Section	No mutation present	Mutation not detected	NA
**31**	FFPE Section	No mutation present	Mutation not detected	NA
**32**	FFPE Section	No mutation present	Mutation not detected	NA
**33**	FFPE Section	c.1799T>A p.(V600E)	V600E mutation detected	NA
**34**	FFPE Section	No mutation present	Mutation not detected	NA
**35**	FFPE Section	No mutation present	Mutation not detected	NA
**36**	FFPE Section	No mutation present	Mutation not detected	NA

**Fig 2:**
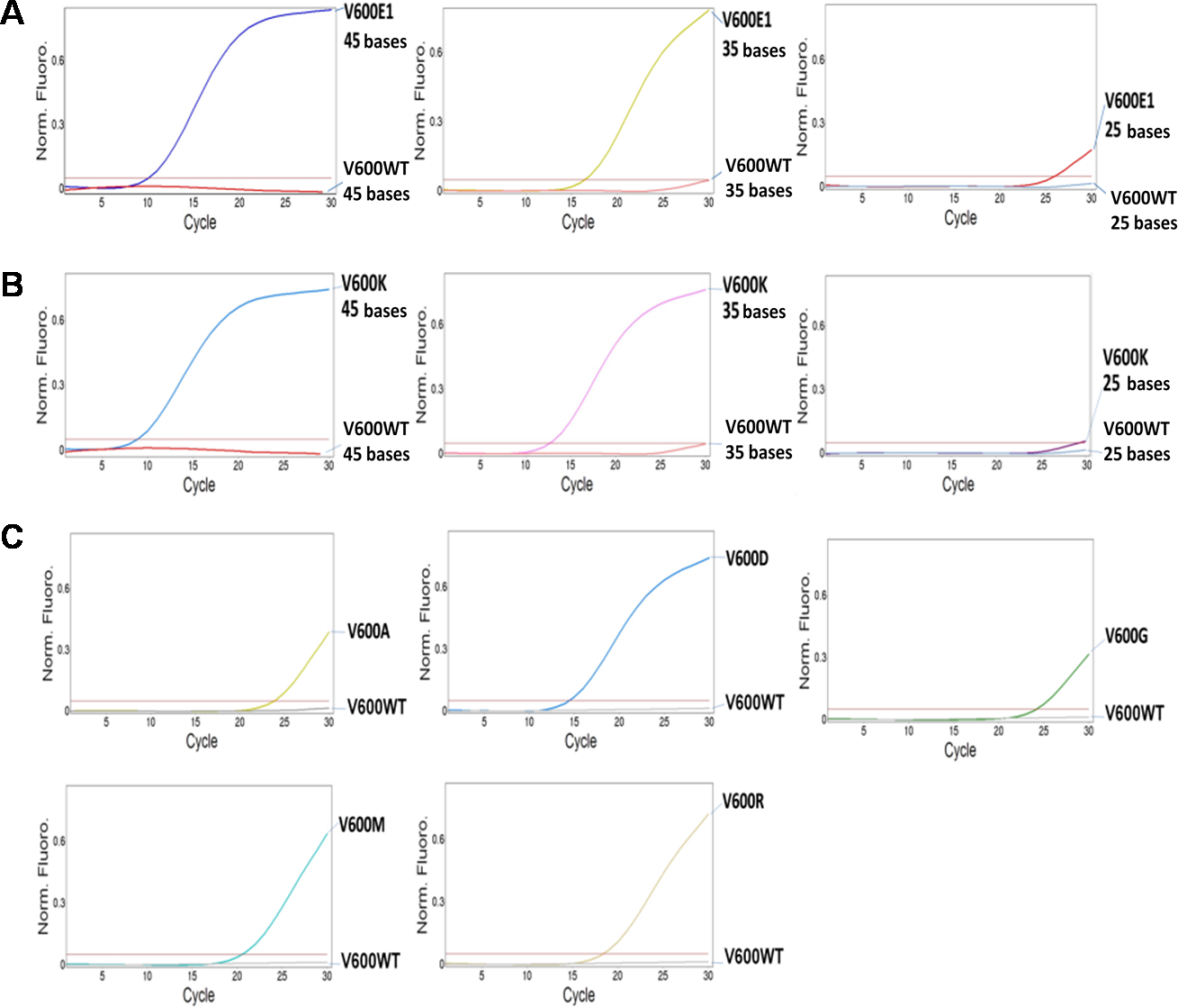
Identification of *BRAF* mutations in ultra-short DNA. A and B. Detection of BRAF V600E1 mutation (A) and V600K mutation (B) in 45-, 35-, or 25-base synthesized oligonucleotides (S1 Table), which mimic severely fragmented single-stranded DNA. C. Cross-reactivity with non-target mutations, i.e. BRAF V600A, V600D, V600G, V600M, and V600R, presented in 45-base oligonucleotide templates (S1 Table). Allele-specific primers for BRAF V600E and V600K were combined in the same quantitative PCR reaction.

The accuracy of PCR-based diagnostic assays depends greatly on the DNA quality of specimens. Targeting short genomic DNA region will ensure that our assay efficiently detect mutations in clinical specimens containing highly fragmented genomic DNA. We examined the ability of our assay to identify *BRAF* mutations in shorter DNA fragments (Fig 2A and 2B). Although BRAF V600E1 or V600K mutations in 35-base templates were detected with relatively delayed amplification as compared to detection of mutations in 45-base templates but it is still clearly to distinguish mutant templates from templates with wild-type alleles. Mutations in 25-base templates could not be amplified by this AS-PCR assay. We evaluated specificity of *BRAF* mutation detection and found that our assay can detect *BRAF* V600D (c.1799_1800delTGinsAT) mutations (Fig 2C). The *BRAF* V600M (c.1798G>A) and V600R (c.1798_1799delGTinsAG) mutations were also picked up with less efficiency while *BRAF* V600G (c.1799T>G) and V600A (c.1799T>C) mutations were barely amplified. No amplifications were found in any of the wild-type templates.

### Thai colorectal cancer tissues submitted for *KRAS* mutation analysis contain poor-quality DNA

We examine our assay performance using DNA extracted from FFPE cell block sections used for EQA schemes which mimic processed tissue sample quality. For 30 samples tested, six samples have *BRAF* V600E mutant alleles (34–66%) and the rest of FFPE samples contain only *BRAF* wild-type cells. Mutation analysis results were shown in Table 1. Neither false-positive nor false-negative results were obtained in our PCR assays in comparison with well-established droplet digital PCR assay results.

**Fig 3:**
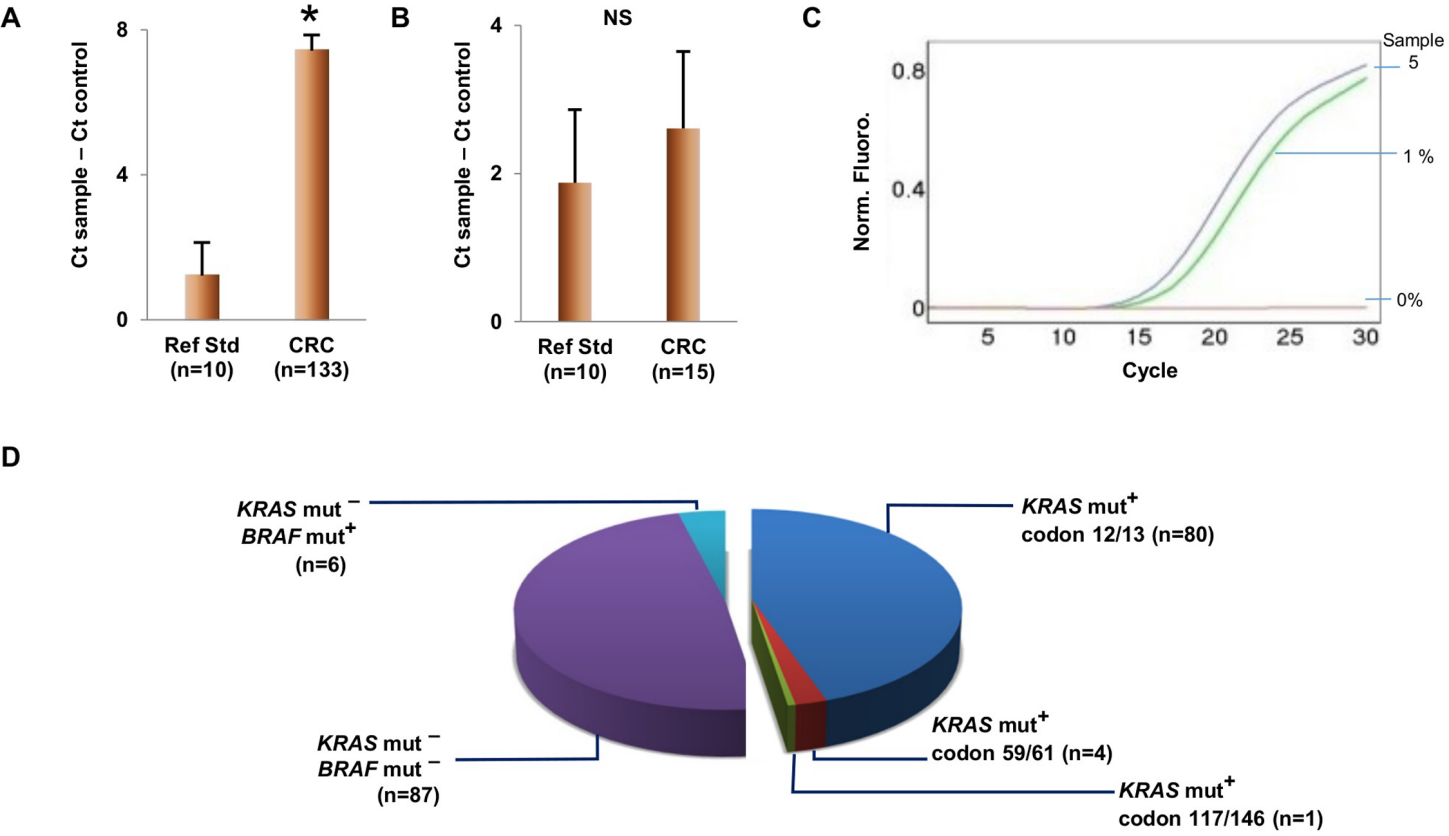
DNA sample quality and mutation status of study samples. A and B. Assessment of 200-bp (A) and 41-bp (B) amplifiable DNA in study samples. DNA samples isolated from cell line (control), FFPE reference standards and FFPE CRC tissues were evaluated. We included the control samples in every run to allow comparisons between runs. The amount of amplifiable DNA was compared to control by comparing the cycle thresholds (Ct) obtained from the quantitative PCR results. Error bars indicate the standard error of the mean. * = statistically significant t-test result (p< 0.01), NS = not statistically significant. Ref Std = FFPE reference standards, CRC = colorectal cancer tissue specimens, Ct = cycle threshold. C. Detection of *BRAF* mutation in poor-quality DNA obtained from FFPE CRC tissue (Sample 5 in Table 3). Genomic DNA sample from HEK-293 cells were used as negative control. D. *KRAS* and *BRAF* mutation status of 178 FFPE CRC tissues. The samples analyzed by methods other than cobas® KRAS Mutation Test, AmoyDx® *KRAS* Mutation Detection Kit and our AS-PCR assay were excluded from this study. mut^-^ = mutation not detected, mut^+^ = mutation detected, ND = not determined.

In our study, DNA quality is demonstrated by amplification of 200-bp target using quantitative PCR (qPCR), the most suitable technique for checking the quality of FFPE-DNA. PCR amplification is affected by the quality of DNA samples. The FFPE reference standards (cell block sections) contain good quantities of amplifiable DNA with the length of fragments of 200 nucleotides (Fig 3A). Our DNA samples from FFPE CRC tissues contain significantly low amplifiable DNA as compared to those from FFPE cell block sections (p-value < 0.01). We further selected 15 FFPE CRC tissue samples with very low amplifiable DNA assessed by 200-bp qPCR assay for amplification of a 41-bp region. We found that the Ct values of these samples are not different from those of FFPE reference standards (Fig 3B). These results suggest that amplification of longer targets was greater affected and confirm that the quality of DNA samples is poor.

Our laboratory mainly used cobas® KRAS Mutation Test or AmoyDx® *KRAS* Mutation Detection Kit to provide a *KRAS* mutation testing service. All of our 178 colorectal cancer specimens tested by these techniques in the years 2013–2017 are FFPE tissues. Three-fifths (106 samples) of 178 FFPE specimens are from male patients (Table 2). Twenty nine specimens (16.3%) were taken from the patients aged over 70 years. Nearly one-fifth (32 samples) of the tissues was collected from metastatic sites. The most common metastatic sites are liver (12 samples) and lung (9 samples). Percentage of neoplastic cells in these specimens examined by pathologists varies from 5–100%.

**Table 2:** Gender, age, and tumor location distribution of cases used in *KRAS* and *BRAF* mutation analysis.

	Characteristics	*KRAS* mutation analysis				*BRAF* mutation analysis			
		n	mut^+^	%	p-value	n	mut^+^	%	p-value
**Gender**	Male	106	48	45.3	0.448^a^	58	4	6.9	1.000^a^
	Female	72	37	51.4		35	2	5.7	
**Age**	≤70	149	66	44.3	0.036^b^	83	6	7.3	1.000^a^
	>70	29	19	65.5		10	0	0.0	
**Tumor location**	Colorectal tissues	146	65	44.5	0.065^b^	81	5	6.2	0.574^a^
	Metastatic tissues	32	20	62.5		12	1	8.3	

^a^Fisher’s exact test

^b^Chi-squared test

The lower limits of detection of cobas® KRAS Mutation Test and AmoyDx® *KRAS* Mutation Detection Kit established at our laboratory using DNA reference standards were 1% *KRAS* mutant alleles (S3 and S4 Tables). The overall frequency of *KRAS* mutation detected by either assay in Thai FFPE CRC tissues is 47.8% (Fig 3D). Forty-eight percent (75 samples) of 156 DNA samples analyzed by cobas® KRAS Mutation Test were *KRAS* mutation-positive. Two samples have *KRAS* mutations at codon 61 and the rest of samples contain mutations at codons 12 or 13. The *KRAS* mutations were detected by AmoyDx® *KRAS* Mutation Detection Kit in 10 of 22 DNA samples (45.5%). Three samples contain *KRAS* mutations at codons 59, 61, 117, or 146. We found no significant association between *KRAS* mutation status and sex or tumor location (metastatic versus primary tumors) (Table 1). The *KRAS* mutation rate is significantly high in CRC patients aged over 70 (65.5%) (p-value = 0.036).

### Small subgroup of Thai *KRAS* mutation-negative colorectal cancers harbor *BRAF* V600 mutations

*KRAS* and *BRAF* mutations were nearly mutually exclusive [36]. We performed *BRAF* mutation analysis using our ultra-short allele-specific PCR assays in FFPE DNA samples that *KRAS* mutations were not detected. Ninety-three DNA samples used for further *BRAF* mutation detection have similar characteristics to 178 samples analyzed for *KRAS* mutation. Ten out of 93 FFPE specimens (10.8%) were taken from the patients aged over 70 years. Approximately three-fifths (58 samples) of specimens are from male patients. Thirteen percent (12 samples) of the tissues was collected from metastatic sites. The most common metastatic site is liver (7 samples).

Mutations of *BRAF* gene were detected in 6 samples (6.5%). Information on these cases was shown in Table 3. Mutated *BRAF* gene was found in 4 male (6.9%) and 2 female (5.7%) patients. All patients except one were over 50 years of age. *BRAF* mutation-negative CRC were not included in this table. Detection of *BRAF* V600E mutation in one of those specimens is demonstrated in Fig 3C.

**Table 3:** Characteristics of *BRAF* V600E mutation-positive cases in Thai colorectal cancer tissues.

Sample number	Age	Sex	Organ	Tumor percentage	qPCR dCt		*BRAF* genotype by GeneRead QIAact Actionable Insights Tumor Panel
					200-bp assay	41-bp assay	
1	58	F	Urinary bladder	NA	11.53	2.12	c.1799T>A mutation not detected
2	63	M	Colon	NA	6.26	1.94	c.1799T>A mutation (16.5%)
3	69	M	Sigmoid colon	80	4.02	0.71	c.1799T>A mutation (18.6%)
4	70	M	Colon	90	4.96	2.36	c.1799T>A mutation (43.0%)
5	28	F	Colon	50	9.76	3.55	c.1799T>A mutation (19.5%)
6	63	M	Ilium	70	2.72	2.79	c.1799T>A mutation (28.9%)

(NA = not available, dCt = Ct _sample_−Ct _control_ in the same run)

We performed sequencing on the samples that *BRAF* mutation was detected by our assay using the GeneReader next generation sequencing system and GeneRead QIAact Actionable Insights Tumor Panel. The average amplicon size for this panel is 134 bp. The *BRAF* V600E mutation was found in five out of six samples. It is noteworthy that PCR amplification of 200-bp fragment of a discordant sample (sample 1 in Table 3) is barely detectable, while the overall quantity of amplifiable DNA is not low as shown by amplification of 41-bp DNA fragments. This finding suggests that this DNA sample is highly fragmented. It is possible that *BRAF* mutation is present in short DNA fragments in this sample.

## Discussion

Identification of the mutation status in cancers becomes mandatory on the best way to guide treatment decision in the era of precision medicine. Although Sanger sequencing can be used to identify somatic mutations in DNA from clinical samples, but this well-established techniques can give false detection as a result of relatively poor analytical sensitivity. It reliably detects mutations at a relative concentration of 20–30% [37]. Another problem with this technique is that the test turnaround time is relatively slow. Pyrosequencing and HRM offer better detection limits with shorter assay times but they still suffer from a limited sensitivity for mutant alleles lower than 5 to 10% [23] which are occasionally found in cancer specimens. At this level of LOD, these techniques are not good choices for mutation detection in plasma and body fluids [38]. Lower limit of detection of at least 1% mutant alleles has been generally recommended for liquid biopsy [39]. Allele-specific PCR (AS-PCR) is another commonly used method for the analysis of clinical specimens harboring somatic mutations of oncogenes. This method relies on mutation-specific primers to discriminate wild-type and mutant alleles. Well-designed and optimized AS-PCR assays generally provide ultrasensitive detection of somatic mutations [40].

The BRAF RGQ PCR and cobas® 4800 BRAF V600 Mutation Test are also based on allele-specific quantitative PCR. The BRAF RGQ PCR claims to detect V600E1, V600E2 and V600K mutant DNA in a wild-type DNA background down to 1.82, 4.31 and 4.34% levels, respectively, when medium input DNA is used. The lower limit of detection of cobas® BRAF V600 test, the US FDA-approved test as companion diagnostic tool, to detect *BRAF* V600E mutation on FFPE tissues is 4.4% mutant alleles when 31.25 ng of DNA are used. However this test failed to detect *BRAF* V600E mutation in the sample that theoretically contained 10% mutant alleles in one study [41]. In our hand, the lowest amount of BRAF V600E mutation that cobas® 4800 BRAF V600 Mutation Test can detect in reference standard DNA is 15% mutant alleles. It has been shown that this assay was able to detect artificial tumor blends with 5% mutant *BRAF* alleles [42]. Further study is needed to determine exact detection limit of this method. Our *BRAF* mutation assays reproducibly detect at least 1% *BRAF* c.1799T>A and c.1798_1799delGTinsAA mutant alleles mixed in wild-type background per 12 ng of genomic DNA. More extensive comparison between assays in reference standard DNA and FFPE samples, and clinical specimens are required to conclude that one assay is better than another.

Targeting short size of the DNA is beneficial to the assay performance. It improves PCR efficiency, minimizes capacity of melanin to interfere with PCR [43] and increases possibility to reveal mutations especially in highly fragmented DNA. The cobas 4800 BRAF V600 Mutation Test targets 116-base pair sequence of the *BRAF* gene. Our assays were designed to target DNA fragments which are shorter than 50 nucleotides. *BRAF* V600E and V600K mutations presented in single-stranded DNA with the size from 35 to 45 bases in length can be identified by our AS-PCR.

The BRAF RGQ PCR contains 4 assays for *BRAF* V600E, V600D, V600K, and V600R mutations. Although cross-reactivity may occurs between mutation reactions, mutations identified by BRAF RGQ PCR can be distinguished. The cobas® 4800 BRAF V600 Mutation Test is not able to distinguish between mutations V600E and V600K. It may also detect other non-V600E mutations such as V600D [43]. Our method has cross-reactivity with BRAF V600D, V600M, and V600R mutations.

Immunohistochemistry (IHC), technique involves antigen–antibody reactions, is widely used in many laboratories. The recently developed monoclonal VE1 antibody has permitted the use of IHC to assess BRAF V600E mutation status in the FFPE tissue in cancers [44–46]. This method may have advantages over PCR amplification in specimens that have been formalin-fixed for long periods [47]. Specificity and cross-reactivity of this antibody are currently being investigated to ensure appropriate interpretation of staining results [48, 49]. This method can be a screening tool for *BRAF* V600E mutation as it allows the detection of small number of cancer cells carrying mutation. Analyzing *BRAF* V600E mutation in circulating melanoma cells by IHC is also possible [50]. Immunohistochemical cross reactivities with *BRAF* V600R and V600K mutations have been reported [23, 51]. False negative in IHC occurs in cases of double mutations in codon 600 and codon 601 of the *BRAF* gene [23, 52]. In contrast, our AS-PCR assays can detect the presence of *BRAF* V600E mutation when it coexists with mutations in codon 601.

In this study, we analyzed *BRAF* mutations in the total of 93 clinical specimens. We report here that *BRAF* V600 mutations are found at approximately 6.5% (6 out of 93) in Thai colorectal cancer patients with no *KRAS* mutations detected in codons 12, 13, 59, 61, 117 or 146. Mutations of *BRAF* gene in *KRAS* mutation-negative CRC tissues have been studied in other Asian patients, including Chinese, Taiwanese, Japanese, and Korean [8–11]. The prevalence rates of *BRAF* V600 mutations in these populations vary from around 1% to 10%.

The DNA present in formalin-fixed material is generally fragmented [53]. This study provides valuable insight into the quality of DNA used for mutation analysis. Samples isolated from our patients’ colorectal cancer tissues contain significantly less amplifiable DNA than samples obtained from FFPE reference standards. This is likely due to differences in a number of specimen attributes, such as fixation time, size of tissues, degree of necrosis and specimen age. The implementation of sensitive assays becomes crucial in this scenario. To investigate *KRAS* mutation status, we used cobas® KRAS Mutation Test and AmoyDx® *KRAS* Mutation Detection Kit which are among the most sensitive methods available in clinical molecular laboratories. This study support the claimed detection limits of <5% and 1–5% mutant DNA in a background of wild-type DNA for cobas® KRAS Mutation Test and AmoyDx® *KRAS* Mutation Detection Kit respectively.

To the best of our knowledge, this is the first report investigating mutations by highly sensitive *KRAS* and *BRAF* mutation assays in Thai CRC tissues. Two reports have been published on the prevalence of *BRAF* mutations at codon 600 in Thai CRC patients. The first study performed Sanger sequencing to analyze 133 frozen tissues and 67 FFPE tissues [54]. No *BRAF* V600 mutation was detected in any of the specimens, while overall incidence of *KRAS* mutations was 23%. This prevalence of *KRAS* mutations in these patients from southern Thailand is quite low compared to our finding (44%). It is likely caused by a difference in detection limits of techniques. It should be highlighted that the LOD of Sanger sequencing for mutations of both *BRAF* and *KRAS* genes at their laboratory and the estimation of tumor cell percentage were not mentioned. Another study utilized two-round allele-specific PCR and high sensitivity DHPLC to analyze 211 specimens obtained from newly diagnosed CRC patients [55]. *BRAF* V600E mutation has been reported in 10.9% of these Thai CRC patients. Although they use sensitive assay with a detection limit of at least 0.5% V600E tumor cells, the copy number of mutant versus wild-type alleles in their cells, cross-reactivity with non-target *BRAF* mutations, and *KRAS* mutation status were not clearly described.

The frequency of *KRAS* mutations in this study appears similar to that in two recent studies in Thai FFPE CRC tissues. Overall prevalence of *KRAS* codon 61 mutation detected by pyrosequencing in one study was 1.7% [56]. In another study, the researchers also used pyrosequencing and found *KRAS* exon 2 mutations in 120 of 270 specimens (44.4%) [57]. They found no association between mutation status and age, sex, or tumor location. Prevalence of *KRAS* mutations in our CRC patients aged over 70 years is higher than that in the rest of study population (p-value = 0.036).

Our simple mutation detection assay may help facilitate the studies in the emerging field of 'molecular pathological epidemiology', which investigate interactive effects of various factors and tumor molecular changes [58, 59]. Integrative analyses of the presence of somatic mutations and exposures (e.g. diet, alcohol, smoking, drug, microbiota, etc.) or intermediate variables are clearly useful for comprehensive understanding of cancer, optimization of cancer prevention and therapy. It has recently been shown that alcohol intake is associated with an increased CRC risk in patients whose tumors do not have a mutation in the *BRAF* gene. This finding suggests that alcohol may have restricted effects on development of CRC cells originating via specific molecular pathways [60]. Another study demonstrated that CRC patients with *BRAF* or *KRAS* mutated tumors were more likely to have an elevated serum level of carbohydrate antigen (CA19-9) and carcinoma embryonic antigen (CEA) [61]. Mutation status of *BRAF* or *KRAS* genes may enhance an important role of these blood biomarkers for precise risk stratification of CRC patients.

In summary, assessment of *BRAF* mutation status in existing DNA from CRC samples that lack KRAS activating mutations by AS-PCR is technically easy and economically favorable. Ultra-short PCR assay with mutation-specific forward primers was proved to be feasible in detecting low abundant *BRAF* V600 mutations in various conditions of DNA samples, i.e. intact genomic DNA, short single-stranded DNA, and DNA from FFPE sections. Molecular assay with specific primers targeting short DNA fragments is therefore particularly useful tool for mutation analyses in a range of clinical matrices, including poor-quality tissues, effusion, plasma and urine.

## Supporting information

S1 Table Oligonucleotides used in this study. (TIF)

S2 Table Primer sets for quantitative PCR. (TIF)

S3 Table Determination of detection limit of cobas® KRAS Mutation Test. (TIF)

S4 Table Determination of detection limit of AmoyDx® *KRAS* Mutation Detection Kit. (TIF)
