# Supplementary figures and images for: Development of ultra-short PCR assay to reveal BRAF V600 mutation status in Thai colorectal cancer tissues
Source: PLoS One. 2018 Jun 7;13(6):e0198795. doi: 10.1371/journal.pone.0198795 (PMC5991739; doi:10.1371/journal.pone.0198795)

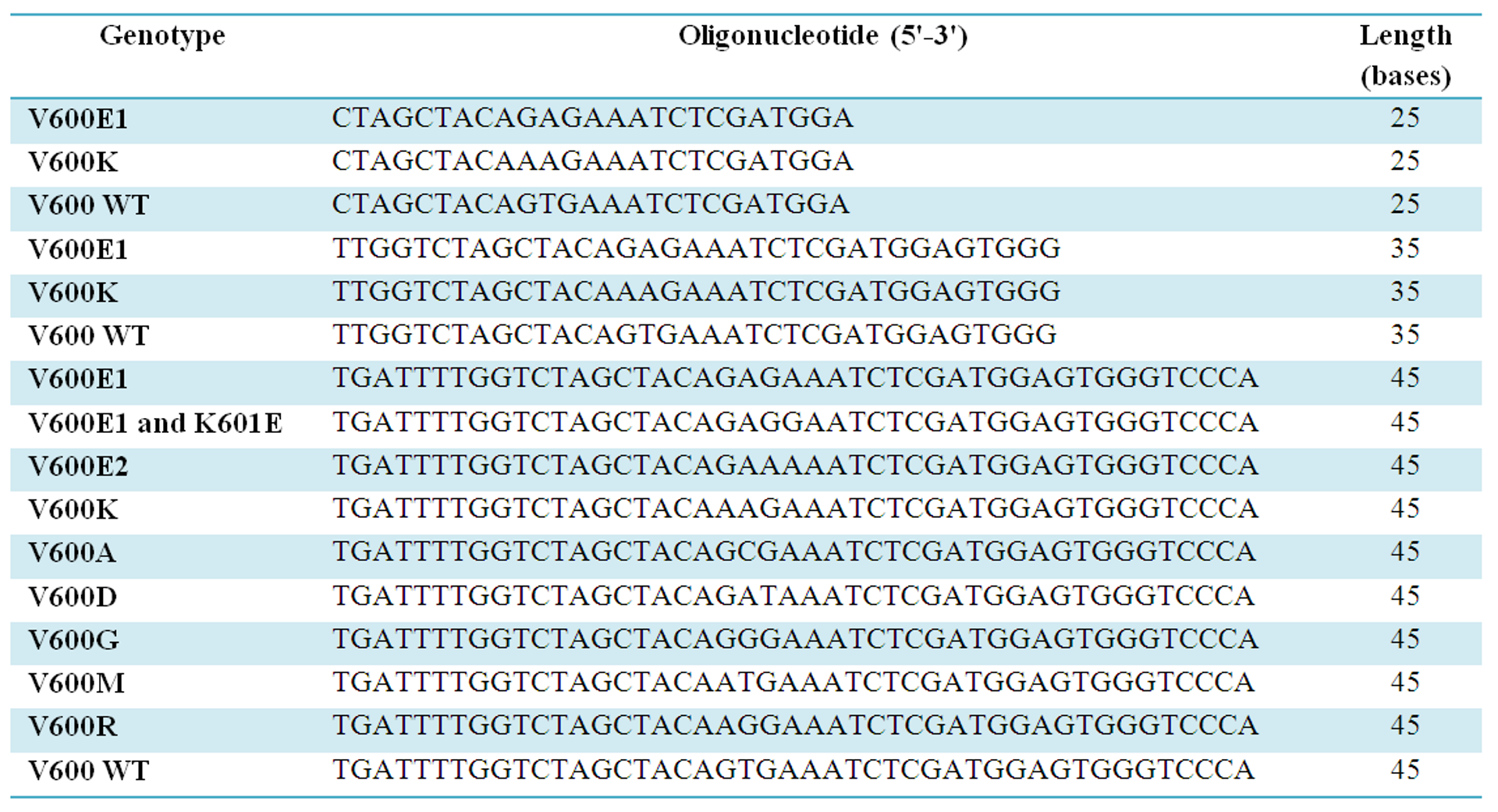

Supplement: S1 Table — (TIF) [file pone.0198795.s001.tif]

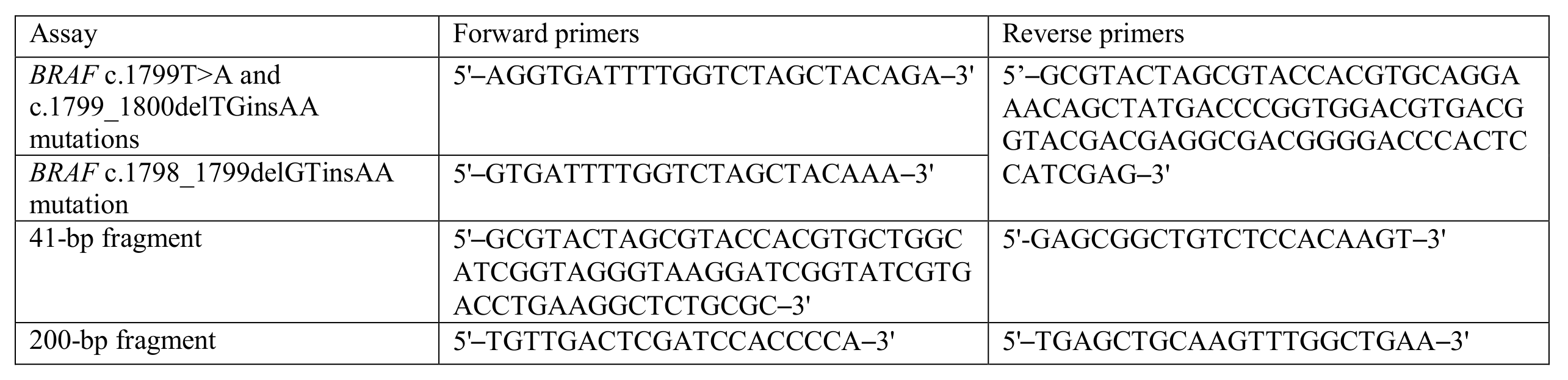

Supplement: S2 Table — (TIF) [file pone.0198795.s002.tif]

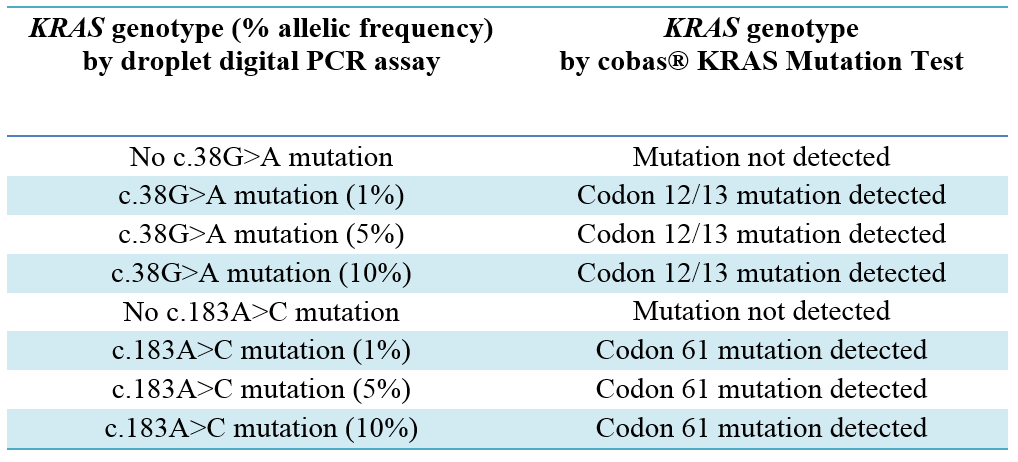

Supplement: S3 Table — (TIF) [file pone.0198795.s003.tif]

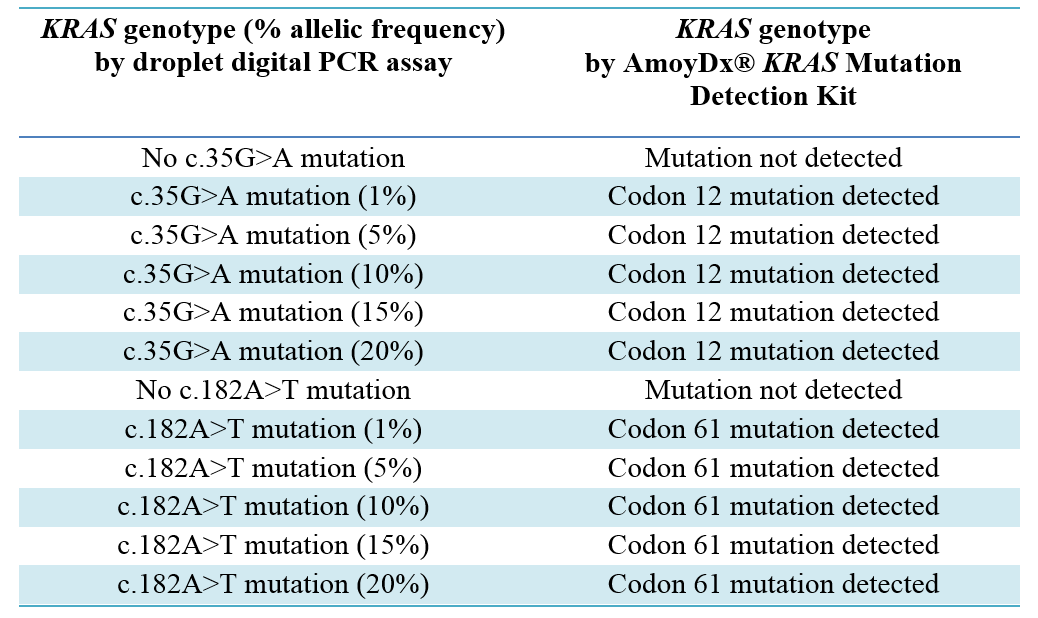

Supplement: S4 Table — (TIF) [file pone.0198795.s004.tif]
